# Laughing Rats Are Optimistic

**DOI:** 10.1371/journal.pone.0051959

**Published:** 2012-12-26

**Authors:** Rafal Rygula, Helena Pluta, Piotr Popik

**Affiliations:** 1 Department of Behavioural Neuroscience and Drug Development, Institute of Pharmacology Polish Academy of Sciences, Krakow, Poland; 2 Faculty of Health Sciences, Collegium Medicum, Jagiellonian University, Krakow, Poland; Université Pierre et Marie Curie, France

## Abstract

Emotions can bias human decisions- for example depressed or anxious people tend to make pessimistic judgements while those in positive affective states are often more optimistic. Several studies have reported that affect contingent judgement biases can also be produced in animals. The animals, however, cannot self-report; therefore, the valence of their emotions, to date, could only be assumed. Here we present the results of an experiment where the affect-contingent judgement bias has been produced by objectively measured positive emotions. We trained rats in operant Skinner boxes to press one lever in response to one tone to receive a food reward and to press another lever in response to a different tone to avoid punishment by electric foot shock. After attaining a stable level of discrimination performance, the animals were subjected to either handling or playful, experimenter-administered manual stimulation – tickling. This procedure has been confirmed to induce a positive affective state in rats, and the 50-kHz ultrasonic vocalisations (rat laughter) emitted by animals in response to tickling have been postulated to index positive emotions akin to human joy. During the tickling and handling sessions, the numbers of emitted high-frequency 50-kHz calls were scored. Immediately after tickling or handling, the animals were tested for their responses to a tone of intermediate frequency, and the pattern of their responses to this ambiguous cue was taken as an indicator of the animals' optimism. Our findings indicate that tickling induced positive emotions which are directly indexed in rats by laughter, can make animals more optimistic. We demonstrate for the first time a link between the directly measured positive affective state and decision making under uncertainty in an animal model. We also introduce innovative tandem-approach for studying emotional-cognitive interplay in animals, which may be of great value for understanding the emotional-cognitive changes associated with mood disorders.

## Introduction

Laughter is a pervasive part of our lives and important component of human nature, but contrary to popular opinion, laughter is not unique to humans [1]. From at least the time of Darwin [2], it has been known that chimpanzees and other great apes also perform a laugh-like vocalization. Recent research revealed that laughter may not even be a phenomenon limited to primates. Also in rats, the 50-kHz Ultrasonic Vocalisations (USVs) have been postulated to reflect a positive affective state akin to primitive human joy and laughter [3], [4]. Indeed, reports from the past decade have yielded enough experimental evidence about the similarities between rat 50 kHz USVs and human laughter, to realistically hypothesize that they are neurally and functionally homologous at behavioural and physiological levels. It has been shown for instance that the 50 kHz USVs in rats are uniquely elevated by hedonic stimuli [5]–[10] and suppressed by aversive stimuli [8], [11]. Rates of 50 kHz USVs were positively correlated to the rewarding value of eliciting stimulus [5] and playbacks of these vocalizations were rewarding. The neural and pharmacological substrates of 50 kHz USVs were also consistent with those of human positive affective states; μ-opiate and dopamine agonists, as well as electrical brain stimulation of the mesolimbic dopamine system, increased rates of 50 khz USVs in rats [6], [10]. The 50 kHz ultrasonic chirping was evident during the anticipatory phase of rat sexual behaviour [12] and anticipation of rewarding brain stimulation [6], during the positive social interchange of rough and thumble play [13] and during playful, experimenter administered manual somatosensory stimulation – tickling [3], [14]. Of all manipulations that elicit 50 kHz chirps in rats, tickling by human elicits the highest rate of these callings [14], providing therefore a tool for modelling and measuring positive affective states in experimental animals and for studying the laughter itself.

In humans cognitive processing can be biased by emotions. Specifically, negative affect is associated with increased expectation of punishment, greater attention to potential threats and a tendency for pessimistic judgement of ambiguous stimuli [15], [16]. Conversely, positive emotions are associated with greater optimism [17]. The same becomes evident for animals. Since publication of the seminal study by Harding et al. [18] showing existence of the stress induced judgement bias in rats, the affect contingent, cognitive biases have been described in a range of species, including rats [19], dogs [20] rhesus macaques [21], sheep [22], chicks [23], starlings [24] and honeybees [25]. However, there have been no studies, to date, in which the valence of animal emotions associated with such cognitive biases, would not only be assumed but objectively measured.

Here we present the results of an experiment where the affect-contingent judgement bias has been produced by objectively measured positive emotions. We trained rats in operant Skinner boxes to press one lever in response to one tone to receive a food reward and to press another lever in response to a different tone to avoid punishment by electric foot shock [19]. After attaining a stable level of discrimination performance, the animals were subjected to either handling or experimenter-administered tickling. This procedure has been confirmed to induce a positive affective state in rats, and the 50-kHz ultrasonic vocalisations (rat laughter) emitted by animals in response to tickling have been postulated to directly index positive emotions akin to human joy. During the tickling and handling sessions, the numbers of emitted high-frequency 50-kHz calls were scored. Immediately after tickling or handling, the animals were tested for their responses to a tone of intermediate frequency, and the pattern of their responses to this ambiguous cue was taken as an indicator of the animals' optimism.

## Results and Discussion

To directly link affective state with cognitive bias, we combined two innovative but validated behavioural techniques for the induction, objective measurement and evaluation of cognitive outputs of emotions in animals. To induce positive emotions, the animals were subjected to playful, experimenter-administered, manual, somatosensory stimulation-tickling [28]. To directly index positive affect in rats, we have measured the tickling-induced 50-kHz ultrasonic vocalisation (rat laughter) that has been postulated to be a correlate of human joy [28]. For the evaluation of the affect-contingent cognitive bias, we have used the ambiguous-cue interpretation paradigm [19] where animals must decide about the valence of ambiguous stimuli. We report that directly measured positive emotions manifested by laughter can trigger optimistic judgement bias in rats.

**Figure 1:**
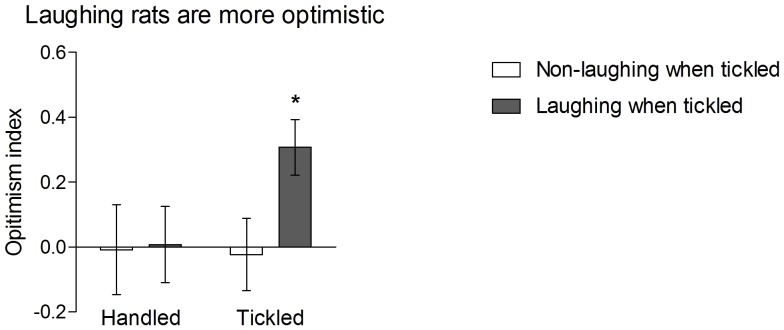
Laughing rats are optimistic. Mean ± SEM optimism index (see text for details) of the “Laughing when tickled” (filled bars, N = 13) and “Non-laughing when tickled” (open bars, N = 13) groups of rats measured after handling and tickling. The optimism index above 0 indicates an overall positive judgement and optimistic interpretation of the ambiguous cue. * indicates significant (p≤0.05) differences between the “Laughing when tickled” and “Non-laughing when tickled” groups.

Initial analysis revealed no significant differences in the responses to the ambiguous cue between the tickled and handled groups of animals (t_(25) = _-1.29, NS). However, when based on the number of emitted 50-kHz USVs (ranging from 0 to 134), the rats were divided into 2 sub-groups using a median split, 2-way repeated measures ANOVA performed on the optimism index data revealed a significant Group×Tickling interaction (F_(1,24) = _6.41, p = 0.018). The animals that responded to tickling with a high (>21 per tickling session) number of 50-kHz USVs (the “Laughing when tickled” group) were more optimistic in the subsequent ambiguous-cue interpretation tests (Figure 1) than the animals that hardly vocalised or did not vocalise at all in response to tickling (the “Non-laughing when tickled” group) (p = 0.019, Sidak test) and were more optimistic after tickling than after handling (p = 0.010, Sidak test). The simple main effects of the Group (F_(1,24) = _1.05) and Tickling (F_(1,24)_ = 2.04) were not significant. The average number of 50-kHz USVs emitted during a 30-s tickling session by the “Laughing when tickled group” was 60±9, while the average for the “Non-laughing when tickled” group was 5±2. Control-handled animals did not emit any measurable calls.

Previous studies have demonstrated that the behavioural effects of tickling are strongly dependent on the number of emitted 50-kHz USVs. For instance, adolescent rats that emit abundant 50-kHz tickle-induced USVs exhibited more tickling-induced place preference compared to adults that responded much less to the tickling [5]. In a simple approach to the hand paradigm, the animals that chirped the most in response to tickling exhibited the fastest approach speeds and more readily approached a hand that had tickled them, yielding many 50-kHz USVs, than a hand that had only petted them, yielding no calls [5]. The rates of emitted 50-kHz USVs have also been positively correlated with the reward values of the eliciting stimuli [8], [10].

It has been postulated that the differences in the responses to tickling, with high or low numbers of 50-kHz USVs, relate to the animal's emotional reactivity, with the rats that vocalise being a more sensitive subgroup [29]. Notably, in the study by Mallo et al. [29], the number of tickling-induced 50-kHz USVs was established over 2 consecutive weeks of tickling and only then correlated with subsequent behaviour. In the present study, we measured tickling-induced laughter not as a stable behavioural trait established over several weeks but as a direct indicator of the animals' positive affective state [30].

**Figure 2:**
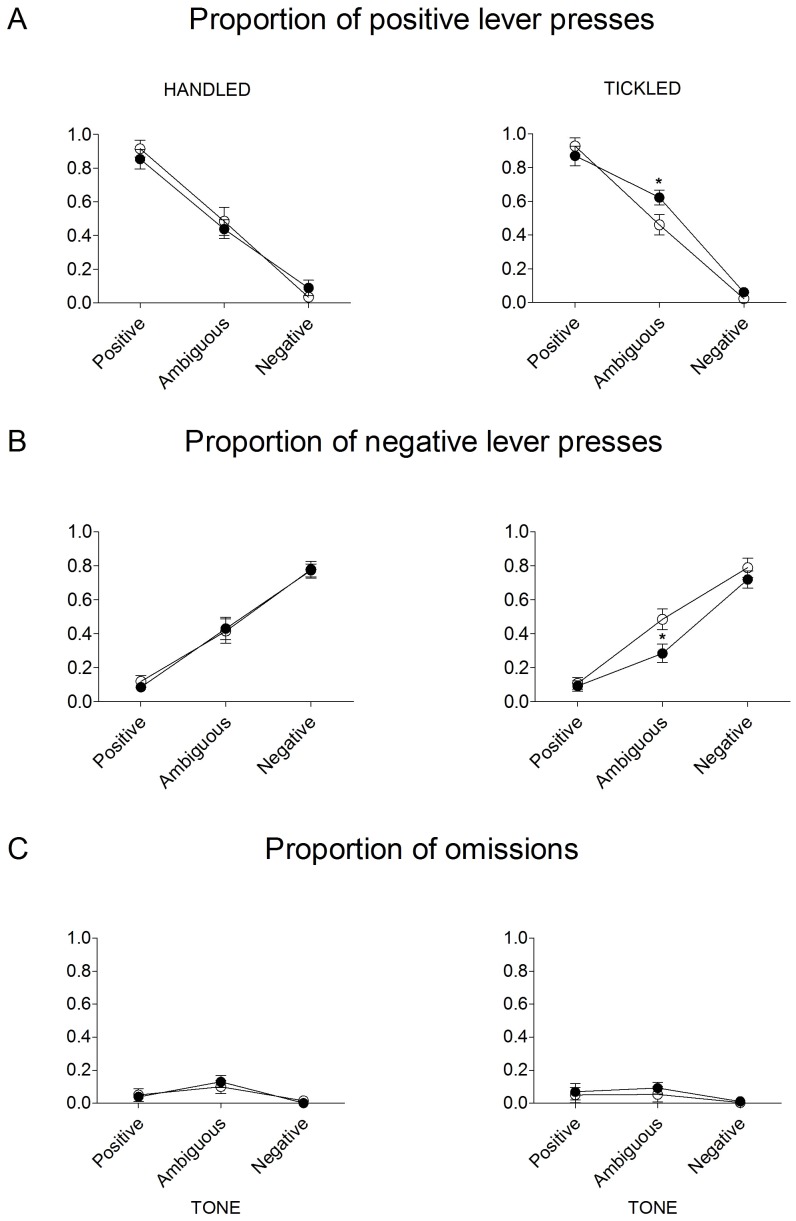
Optimistic bias is specific to the ambiguous cue. Mean ± SEM proportion of positive, negative and omitted responses to the trained and ambiguous tones in the “Non-laughing when tickled” (open circles, N = 13) and the “Laughing when tickled” (filled circles, N = 13) groups of rats after handling and tickling. * indicates significant (p≤0.05) difference between the “Non-laughing when tickled” and the “Laughing when tickled” groups of rats.

As shown on Figures 2A–C, the tickling-induced, positive/optimistic response bias in the “Laughing when tickled” group of rats was specific to the ambiguous, intermediate tone, with no effects of tickling observed in the responses to the unambiguous positive and negative tones (significant Lever×Tickling×Tone×Group interaction (F_(2,48) = _3.49, p = 0.038)). This optimistic bias resulted from increased positive (p = 0.041, Sidak test) and decreased negative (p = 0.019, Sidak test) responses. Four-way repeated measures ANOVA also revealed significant effects of Tone (F_(2,4) = _7.0, p = 0.002), significant Lever×Tone interaction (F_(2,48) = _231.3, p = 0.0001), and significant Lever×Tickling×Group interaction (F_(1,24) = _6.9, p = 0.015). All other effects and interactions were not significant (largest F_(1,24) = _4.64). There were no between-group differences in the proportion of the trials in which the animals did not respond (response omissions) to the ambiguous and unambiguous tones after either handling or tickling. However, all animals demonstrated more omissions in response to the ambiguous tone compared to the trained tones (significant main effect of Tone (F_(2,48) = _10.5, p = 0.001)). All other effects and interactions were not significant (largest F_(2,48) = _2.23).

Although further studies should directly pinpoint the neural correlates of the observed optimistic bias, tickling-induced positive emotions only biased the responses to the ambiguous tone, with unaltered processing of the tones predicting reward and punishment, which suggests the involvement of the neural circuits related to decision making under circumstances of ambiguity, uncertainty, and risk. Those neuroanatomical substrates likely include the anterior cingulate, the parietal and prefrontal cortices [31]–[34] and the amygdala, which has been suggested to play a role in valence representations [35] and in the processing of ambiguity [36]. The optimistic bias that we observed indicates that, after laughing, the rats may have an increased expectation of reward (increased response to the positive lever) and a decreased expectation of punishment (decreased response to the negative lever). These expectations could reflect a change either in their perception of the probability of receiving the reward and punishment or in the impact/value of the reinforcement. Although we cannot exclude the possibility that an altered sensitivity to reward and/or punishment influenced reaction of animals only to the new tone (ambiguous cue), the unaltered responses to the trained tones, together with the unchanged patterns of omission, suggests that the absolute values of reward and punishment remained unaffected by positive emotions. Notably, contrary to previous studies, we did not investigate different degrees of ambiguity [18], [19]. The experimental design with only one ambiguous tone allowed us to apply a reasonably high (10) number of these stimuli in only one testing session, and the 50 discriminations per session provided a broad and precise scale for the valence of responding.

This study is the first to demonstrate an affect-contingent cognitive bias, in which the valence of animal emotion was not assumed but directly evaluated using independent measure. Our findings provide evidence and validate the hypothesis that the assessment of cognitive biases can be used for the measurement of animal emotions. These results also provide an alternative approach for studying emotional-cognitive interplay in an animal model where both components can be independently measured. It is possible that this tandem technique could be adapted for studying the affective changes associated with anxiety or depression. An animal model that provides a measurable positive affect that results in an optimistic bias may be of great value for understanding the emotional-cognitive changes associated with mood disorders.

## Materials and Methods

### Ethics Statement

The experiments were conducted in accordance with the NIH Guide for the Care and Use of Laboratory Animals and were approved by the Ethics Committee for Animal Experiments at the Institute of Pharmacology Polish Academy of Sciences.

### Subjects and housing

Twenty-six male *Sprague–Dawley* rats (Charles River, Germany) weighing 175–200 g upon arrival were used in this study. The rats were group-housed (4 rats/cage) in a temperature (21±1°C) and humidity (40–50%) A/C-controlled colony room under a 12/12-h light/dark cycle (lights on at 06:00 h). In all experiments, the rats were mildly food restricted to approximately 85% of their free feeding weights. This was achieved by providing 15–20 g of food per rat per day (standard laboratory chow). Food restriction started 1 week before the beginning of training. Water was freely available, except during test sessions. Behavioural procedures and testing were carried out during the light phase of the light/dark cycle.

### Apparatus

The behavioural tasks were carried out in 8 computer-controlled Skinner boxes (MedAssociates, St Albans, Vermont, USA), each equipped with light, a speaker, a liquid dispenser (set to deliver 0.1 ml of 20% sucrose solution), a grid floor through which scrambled electric shocks (0.5 mA) could be delivered, and 2 retractable levers. The levers were located at 2 opposite sides of the feeder. All behavioural protocols, including data acquisition and recording, were programmed in MedState notation code (Med Associates) by the authors. The experimental procedures for the ambiguous-cue interpretation test used here were modified versions of the procedures described by Enkel and colleagues [19].

### Behavioural training

#### Positive Tone Training

During this phase, the rats were trained to press the lever located on the left side of the feeder to receive the sucrose solution when a tone (50 s, 2000-Hz at 75 dB sound pressure level (SPL) or 9000-Hz at 75 dB SPL (counterbalanced)) signalled reward availability. Due to its association with a palatable reward, this tone acquired positive valence and is referred to as the ‘positive tone’, and the associated lever is referred to as the ‘positive lever’. Reliable active lever pressing for the reward was achieved in 3 training steps: a.) Presentation of the positive tone (lasting 50 s) co-occurred with constant delivery of the sucrose solution and was followed by a 10 s intertrial interval (ITI); b.) Presentation of the positive tone co-occurred with left lever extension and was followed by a 10 s ITI. Each lever press during the tone was continuously rewarded by sucrose solution delivery and c.) was similar to (b); however, after the first lever press and reward delivery, the tone was terminated and followed by a 10 s ITI. Each training session lasted 30 minutes, and the trainings were continued until the animals attained a stable performance on each of the training steps. Positive tone training was followed by negative tone training.

#### Negative Tone Training

During this stage, the rats were trained to press the lever located on the right side of the feeder to avoid electric shock when another tone (9000-Hz at 75 dB SPL or 2000-Hz at 75 dB SPL (counterbalanced)) signalled forthcoming punishment. Due to its association with concomitant punishment, this tone acquired negative valence and is referred to as the ‘negative tone’, and the associated lever is referred to as the ‘negative lever’. A reliable active lever press avoidance reaction was achieved in 2 training steps: a.) The presentation of the negative tone was paralleled by the occurrence of electric shocks unless the rat pressed the right (negative) lever, terminating the shock and tone presentation, and b.) the presentation of the negative tone preceded the occurrence of electric shocks. The delay from the tone onset to electric shock occurrence was progressively increased from 1 s to 40 s. Pressing the negative lever before shock onset terminated the tone and began a 10 s ITI (prevention response). Pressing the negative lever after the shock onset terminated the tone and shock and is referred to as the “escape response.” The maximum duration of the tone/shock-application was 50 s (i.e., 40 s of tone presentation followed by 10 s of tone/shock co-occurrence) and tone presentations were separated by 10 s ITIs. Daily training sessions consisted of 40 tone presentations. The animals had to accomplish at least 60% correct prevention responses before proceeding to discrimination training.

#### Discrimination Training

During this phase, the rats were trained to discriminate between positive and negative tones by responding to the appropriate levers (as learned in previous stages of the training) to maximise reward and minimise punishment delivery. The tones, 20 positive and 20 negative, were presented pseudo-randomly and separated by 10 s ITIs. Pressing the positive lever during positive tone presentation resulted in instant reward delivery and initiated the ITI. Pressing the negative lever during negative tone presentation resulted in negative tone termination and initiated the ITI. Pressing the wrong lever (e.g., pressing the left instead of the right lever in response to negative tone presentation) as well as escape responses or response omissions were considered failure trials. Animals had to minimally achieve 70% correct responses at each lever to proceed to the ambiguous cue test.

#### Ambiguous Cue Testing

The ambiguous cue testing session consisted of 20 positive, 20 negative, and 10 intermediate (ambiguous) tone presentations. The frequency of the intermediate tones was set to 5000-Hz at 75 dB. This frequency was chosen based upon the protocol described by [19] and confirmed to be intermediate in terms of the response pattern in a pilot experiment (data not shown). The tones were presented in a pseudorandomised order and separated by 10 s ITIs. Any lever press during ambiguous tone presentation terminated the tone but had no consequences. If a rat did not respond during the 50 s of ambiguous tone presentation, the tone was terminated and a response omission was scored.

### Tickling and USVs recording

The animals were investigated for the propensity to produce high-frequency calls in response to playful, experimenter-administered manual somatosensory stimulation – tickling in a room adjacent to the operant conditioning room. Tickling was performed using modification of the method described by Panksepp and Burgdorf [14], consisting of gently holding the rat on its back in the investigator's left hand and rapid right-hand finger movements across the ventral body surface of the animal, followed by its release after 30 s of stimulation. Control animals were held for 30 s in the same way but were not tickled. Ultrasonic vocalisations were audible in headphones using an ultrasound heterodyne bat detector microphone (Ultrasound Advice, UK) that was set to reflect the high-frequency 50-kHz “laughter” calls. The same vocalisations were visualised on-line by a PC computer running Raven Pro 1.4 interactive sound analysis software (Cornell Lab of Ornithology, Bioacoustics Research Program, USA) connected via the A/D converter DAQ device (USB-6251 1.25 MS/s M Series) to another ultrasound microphone (Avisoft, Germany). Thus, the calls were recorded by investigators who simultaneously listened to and scored the vocalisations on the computer screen [26].

### Experimental design and behavioural measures

**Figure 3:**
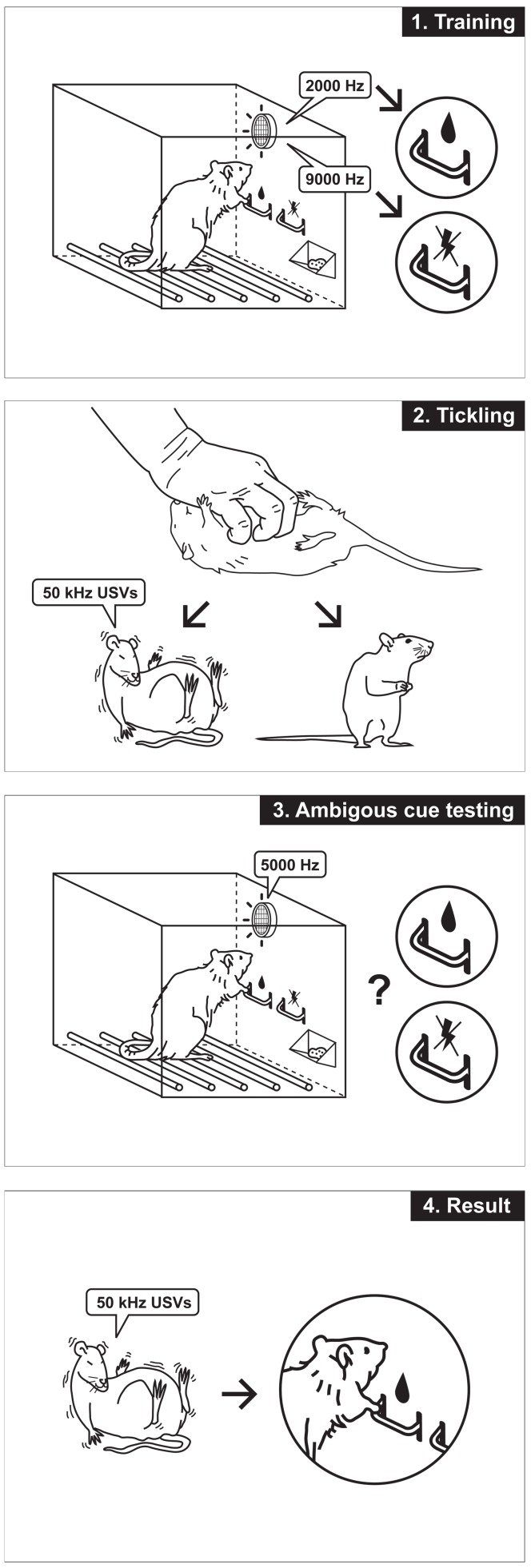
Schematic representation of the experimental schedule.

The experimental design is schematically presented on the figure 3. After attaining a stable discrimination performance (more than 70% correct responses to each tone over 3 consecutive days), each rat was either handled (Sham procedure) or tickled for 30 s in a cross-over design over 2 consecutive days. In this way, each animal served as its own control. After tickling or handling, the animals were immediately subjected to ambiguous-cue testing. During tickling, the number of high-frequency 50-kHz calls was recorded, and the calls were scored. Based on the number of emitted 50-kHz USVs, the tickled rats were divided into 2 sub-groups of “Laughing when tickled” and “Non-laughing when tickled” animals using a median split. These 2 groups were analysed separately. During ambiguous cue testing, the responses to each tone (positive, ambiguous and negative) were scored and analysed as the proportion of the overall number of responses to a given tone. The proportion of omissions was analysed separately. To calculate the optimism index, the negative responses to the ambiguous cues were subtracted from the positive responses, resulting in values between −1 and 1, with values above 0 indicating an overall positive judgement and optimistic interpretation of the ambiguous cue.

### Statistics

The data were analysed using SPSS (version 20.0, SPSS Inc., Chicago, IL, United States). Initial analysis of the effects of tickling on ambiguous tone interpretation was performed using a paired *t*-test. The optimism index data were subjected to 2-way repeated measures Analysis of Variance (ANOVA) with the between-subjects factor of Group (2 levels: “Laughing when tickled” and “Non-laughing when tickled”) and the within-subjects factor of Tickling (2 levels: Handled and Tickled). The effects of tickling on tone interpretation were analysed using 4-way repeated measures ANOVA with the between-subjects factor of Group (2 levels: Non-laughing when tickled and Laughing when tickled) and the within-subjects factors of Lever (2 levels: Positive and Negative), Tickling (2 levels: Handled and Tickled) and Tone (3 levels: Positive, Ambiguous and Negative). Response omissions were analysed separately using 3-way repeated measures ANOVA with the between-subjects factor of Group (2 levels: “Non-laughing when tickled” and “Laughing when tickled”) and the within-subjects factors of Tickling (2 levels: Handled and Tickled) and “Tone” (three levels: Positive, Ambiguous and Negative). For pair-wise comparisons, the values were adjusted using Sidak's correction factor for multiple comparisons [27]. All tests of significance were performed at α = 0.05. Homogeneity of variance was verified using Levene's test. For repeated-measures analyses, the sphericity was also verified using Mauchly's test. The data are presented as the mean ± SEM.
